# Mesenchymal stem cells ameliorate Sjögren disease by suppressing B cells through the Pik3cb/Akt/mTOR pathway

**DOI:** 10.3389/fimmu.2026.1761950

**Published:** 2026-03-12

**Authors:** Zhifang Wu, Chunning Wang, Linsha Ma, Dengsheng Xia

**Affiliations:** 1 Beijing Stomatological Hospital, School of Stomatology, Capital Medical University, Beijing, China; 2 Beijing Friendship Hospital, Capital Medical University, Beijing, China

**Keywords:** autoimmunity, B cells, mesenchymal stem cells, phosphoinositide 3-kinase β, Sjögren disease

## Abstract

Mesenchymal stem cells (MSCs) hold great promise for the treatment of Sjögren disease (SjD) owing to their potent immunomodulatory capacity. However, the precise molecular mechanism by which MSCs regulate the characteristic B cell dysregulation in SjD remains largely unknown. In this study, we found that Pik3cb expression was significantly upregulated in submandibular glands (SMGs) of NOD mice, a well-established SjD model. Notably, genome-wide microarray profiling identified *Pik3cb* as a pivotal mediator of the therapeutic efficacy of allogeneic MSCs in NOD mice, suggesting it plays a role in SjD pathogenesis and treatment. Systematic investigation of the role of Pik3cb in MSC therapy and B cell regulation revealed that MSC administration and pharmacological inhibition of Pik3cb (using TGX-221) significantly attenuated SjD progression. This attenuation was characterised by the robust suppression of B cell responses, including activation, chemotaxis, plasma cell differentiation, and antibody production. Both interventions effectively restored salivary secretion and alleviated lymphocytic infiltration and fibrosis in the SMGs. Concurrently, a significant shift in the cytokine profile was observed, with diminished pro-inflammatory cytokines (IL-4, IL-6, IFN-γ) and upregulated anti-inflammatory factors (IL-10, TGF-β1) in the SMGs and spleens. Additionally, Pik3cb overexpression in B cells abrogated the MSC-induced therapeutic benefits, confirming the specificity of Pik3cb as a target. Finally, mechanistic studies revealed that MSC efficacy was correlated with Pik3cb suppression, resulting in the subsequent downregulation of Akt/mTOR signalling. In conclusion, this study provides mechanistic evidence that MSC therapy mitigates B cell dysfunction in SjD through the Pik3cb/Akt/mTOR pathway. Furthermore, our data identified Pik3cb as a hitherto unrecognized molecular target in SjD pathogenesis, suggesting that its pharmacological inhibition may represent a promising complementary therapeutic avenue for SjD meriting further investigation.

## 1 Introduction

Sjögren disease (SjD), formerly known as Sjögren’s syndrome (1), is a prototypic chronic systemic autoimmune disorder characterised by lymphocyte-mediated destruction and dysfunction of the exocrine glands, primarily affecting the salivary and lacrimal glands (2). Persistent xerostomia (dry mouth) and xerophthalmia (dry eyes) are hallmark clinical manifestations of this syndrome, which substantially affect the patients’ quality of life.

The pathogenesis of SjD involves a complex and dysregulated crosstalk between innate and adaptive immunity (3). Early-stage disease is driven by prominent type I interferon (IFN) signatures, which subsequently induce the overexpression of B cell-activating factor (BAFF). BAFF serves as a crucial link between innate immunity and B cell activation (4) by promoting the survival, differentiation, and autoantibody production of autoreactive B cells (5–7). Concurrently, T-cell polarisation exhibits stage-specific patterns. While Th1 (IFN-γ-producing) and Th17 cells dominate the initial phases, follicular helper T (Tfh) cells play a crucial role in sustaining B-cell hyperactivity during disease progression (8–12). Additionally, the CXCL13/CXCR5 chemokine axis mediates the recruitment of B and Tfh cells to the salivary glands, facilitating the formation of ectopic lymphoid structures (ELS) (13–15). These ELS serve as specialised niches that foster somatic hypermutation and class-switch recombination in B cells, thereby driving the sustained production of high-affinity autoantibodies (16, 17). Ultimately, these cumulative immunological abnormalities lead to progressive infiltration of lymphocytes. Given that B cells play a central role in SjD pathogenesis, they are an important therapeutic target (18).

However, current therapeutic strategies for SjD remain largely limited to symptomatic management (e.g. saliva stimulation), despite significant progress in elucidating SjD pathogenesis (19, 20). Therefore the development of effective disease-modifying therapies is urgently required. Emerging evidence positions mesenchymal stem cells (MSCs) as a promising immunomodulatory therapy for SjD (21). Allogeneic MSC transplantation attenuates glandular lymphocytic infiltration, restores secretory function, and reduces proinflammatory cytokines/autoantibodies in animal models of SjD (22, 23). Additionally, several preliminary clinical trials have suggested the potential therapeutic benefits of MSC transplantation in patients with SjD (22, 24–27). Studies in NOD mice have also shown that MSC administration successfully reduces pathogenic B cell populations, a key pathological driver of SjD. Nevertheless, the specific molecular mechanisms by which MSC therapy directly regulates and corrects B cell dysregulation in the SjD pathological microenvironment remain largely unexplored (20).

Previously, our preliminary genome-wide microarray analysis of the NOD mouse model revealed that Pik3cb and its downstream PI3K/Akt/mTOR pathway are pivotal regulators of MSC-based SjD therapy. Therefore, this study aimed to systematically investigate the role of the PI3K/Akt/mTOR pathway in modulating the therapeutic benefits of MSCs and correcting B cell dysregulation in SjD. We used pharmacological inhibition (using TGX-221) and genetic manipulation (Pik3cb overexpression) to validate target specificity. Additionally, we rigorously assessed the therapeutic outcomes based on functional restoration, immunomodulation, and histopathological repair. Our results will provide insights into novel small-molecule druggable targets for SjD-specific biologics.

## 2 Materials and methods

### 2.1 Animals

Female NOD/Ltj mice (Cdh23ahl, 16 weeks old), a well-established model of spontaneous SjD-like pathology, were used in this study, with age-and sex-matched ICR mice serving as healthy controls. All mice were purchased from Beijing HFK Bioscience Co., Ltd. (Beijing, China) and maintained in a specific pathogen-free animal facility with free access to food and water. All animal experiments and protocols were approved by the Animal Care and Use Committee of the MDKN Biotech (approval no. MDKN-2022-094).

### 2.2 Administration of allogeneic bone marrow-derived MSCs

Allogeneic bone marrow-derived MSCs were obtained as validated and characterised products from Fudan Cell Biotechnology Co., Ltd. (FH-M197; FuHeng, Shanghai, China). Given that females are predominantly affected by SjD, their MSCs may exhibit compromised efficacy. In order to ensure a consistent baseline of immunoregulatory potency and minimize doner-related functional bias, male-derived MSCs were utilized. These cells were originally isolated from the bone marrow of male BALB/c mice (6–8 weeks old) and were characterised using MSC surface markers. MSCs at passage 3 (1 × 10^6^ cells/mouse) were administered to the NOD mice (16 weeks of age) intravenously in 0.15 mL Phosphate-Buffered Saline (PBS) (28). The control animals received an equivalent volume of PBS intravenously.

### 2.3 Pharmacological inhibition of Pik3cb

The Pik3cb-specific inhibitor TGX-221 (S1169, Selleck Chemicals, Houston, USA), was dissolved in 0.5% DMSO (12611S, Cell Signalling Technology, MA, USA). NOD mice in the inhibition group (16 weeks of age) received TGX-221 at a dose of 10 mg/kg (29) via daily intraperitoneal (i.p.) injection. The control group received an equivalent volume of 0.5% DMSO vehicle i.p.

### 2.4 B cell-specific Pik3cb overexpression experiment

An adeno-associated virus 9 (AAV) vector harbouring a B cell-specific promoter (CD19) was used (AAV-CD19-Pik3cb) to achieve B cell-specific Pik3cb overexpression (OE). NOD mice were divided into three groups for this experiment. (i) OE group: received AAV-CD19-Pik3cb only, (ii) OE + MSC group: received AAV-CD19-Pik3cb followed by MSC treatment (for rescue assessment), and (iii) AAV + MSC group: received AAV-CD19-Null (empty vector) followed by MSC treatment (vector control). The mice were anaesthetized, and the AAV suspension (5 × 10^11^ vg/mouse) (30) was injected directly into the spleen glands. One-week post-injection, B cell infection was confirmed by RT-qPCR for *Pik3cb* and immunofluorescence staining for Pik3cb and CD19. Subsequently, mice in the OE + MSC and AAV + MSC groups received an intravenous injection of 1 × 10^6^ MSCs per mouse.

### 2.5 Microarray data acquisition and re-analysis

Transcriptomic profiling of SMGs from the PBS- and MSC-treated NOD mice was performed using the GeneChip Mouse Gene 1.0 ST Array (Thermo Fisher Scientific, USA). Total RNA was extracted from the tissues using TRIzol Reagent (Invitrogen, USA), purified using the RNeasy MinElute kit (Qiagen, Germany). and validated for quality via UV spectrophotometry and denaturing agarose gel electrophoresis. Subsequently, cDNA synthesis, fragmentation, labelling, hybridisation, and signal scanning were conducted following the manufacturer’s protocols. Differential expressed genes (DEGs) were identified based on fold-change (FC) threshold.

To establish the study’s rationale, a targeted re-analysis of the dataset was performed. While broader transcriptomic findings are documented elsewhere, the present re-analysis specifically focuses on the PI3K/Akt/mTOR cascade, identifying the Pik3cb-mediated pathway as a possible central regulatory node in the SMG immune microenvironment.

### 2.6 Saliva flow rate measurement

The salivary secretions were quantitatively assessed using a previously established protocol (28). Briefly, the mice were fasted for 4–6 h (water ad libitum) and mildly anesthetized with ketamine (100 mg/mL; Beijing Double-Crane Pharmaceutical Co., Ltd., China) and xylazine (20 mg/mL; Sigma-Aldrich, St. Louis, MO, USA; 1 μL/g body weight) i.p. Subsequently, saliva secretion was stimulated by administration of pilocarpine solution (0.1 mL/kg body weight, 50 mg/mL; Beijing Double-Crane Pharmaceutical Co., China) i.p. At 2 min post-stimulation, a 75-mm haematocrit tube was carefully placed into the mouse oral cavity and connected to a pre-weighed microcentrifuge tube. Saliva was collected for 10 min and gravimetrically analysed. Results are expressed as saliva flow rate (mg/10 min).

### 2.7 Proteinuria detection

Protein concentrations in the urine samples were determined using a microplate-adapted Bradford assay. Briefly, each urine sample was appropriately diluted, and 20 µL aliquots (in duplicate) were loaded into a 96-well microplate alongside bovine serum albumin standards. Subsequently, 200 µL of Coomassie Brilliant Blue G-250 dye reagent (HY-NE458; Sinouk Institute of Biological Technology, Beijing, China) was added to each well and mixed gently. The plate was incubated at room temperature for 5 min to allow complete colour development. Optical density at 595 nm was measured using a microplate reader (HY-YQ-08; Diatek, Wuxi, China). A standard curve was generated from the absorbance values of known standards, and sample protein concentrations were interpolated from this curve. Quality control samples were included in each run to monitor inter-assay variability.

### 2.8 Histological analysis

The mice were sacrificed 4 weeks post-MSC or TGX-221 administration and the SMGs or spleens were harvested and immediately fixed in 4% paraformaldehyde. The fixed samples were then paraffin-embedded and sectioned into 4 μm sections. The SMG sections were stained with Haematoxylin and Eosin (H&E). The area of inflammatory focus, defined as a lymphoid aggregation of more than 50 lymphocytes per 4 mm^2^, was quantitatively assessed using Image-Pro Plus 6.0 software (16). The percentage of lymphocytic infiltration area was then calculated based on the total area of foci relative to the entire section area. Additionally, Sirius Red staining was performed to evaluate fibrosis in the SMGs. The sections were stained with Sirius Red solution for 20 min and visualised under a polarised light microscope. The type I collagen fibres exhibited a characteristic yellowish-orange or bright red birefringence.

Subsequently, Tyramide signal amplification-based multiplex immunofluorescence staining (TSA) was performed on SMG sections using established protocols to simultaneously detect key markers (31). The primary antibodies (all sourced from Solarbio, Beijing, China) used were rabbit anti-Pik3cb-Cy5 (1:200, K016107P), rabbit anti-CD19-Cy3 (1:500, K003431P), rabbit anti-CD20-FITC (1:200, K016108P), goat anti-IgG-FITC (1:200, SF131), and goat anti- IgM-Cy5 (1:200, K0055G-AF647). The fluorescence signals were quantified using Image-Pro Plus 6.0 software. Percentage of positive area were calculated and defined as the ratio of the antigen-specific fluorescence area to the total tissue area (DAPI-stained area). For each animal, five entire sections of either the salivary glands or the spleen were quantified by an experienced pathologist in a blinded manner (28).

Immunofluorescence staining (IF) of splenic plasma cells (CD138^+^) was performed separately. Sections were heated in Tris-EDTA buffer (pH 9.0, C1037, Solarbio, Beijing, China) for antigen retrieval, followed by incubation with 3% H_2_O_2_ and serum blocking. The sections were incubated overnight with primary antibody rabbit anti-CD138-FITC (1:100, K012955RR, Solarbio, Beijing, China), and secondary antibody goat anti-IgG-FITC (1:200, SF131) for 30 min and washed with PBS three times.

### 2.9 Flow cytometric analysis

The frequency of splenic Tfh and plasma cells was determined using flow cytometry. Briefly, the cells were stained with the following antibodies: anti-mouse CD4-PE/Cy7 (100528; BioLegend, USA), anti-mouse CXCR5-PerCP/Cy5.5 (145508; BioLegend, USA), anti-mouse B220-FITC (103206; BioLegend, USA), and anti-mouse CD138-PE (553714; BD Biosciences, USA). Samples were analysed using the FACS LSR Fortessa (BD Biosciences) and FlowJo software version 10.8 (BD Biosciences).

### 2.10 Enzyme-linked immunosorbent assay

Cytokine and chemokine levels were assessed in tissue lysates extracted from mouse spleen or SMGs. The levels of IL-4, IL-6, IL-10, TGF-β1, IFN-γ, CXCL13, and BAFF were measured by mouse enzyme-linked immunosorbent assay (ELISA) kit (Solarbio, Beijing, China) strictly following the manufacturer’s instructions.

### 2.11 Real-time quantitative PCR

Total RNA was isolated from samples using TRIzol reagent (R1100, Solarbio) and reverse transcribed using an M-MLV Reverse Transcription Kit (RP1105, Solarbio). β-Actin and Gapdh were used as internal controls. Relative gene expression was calculated using the 2^−ΔΔCt^ method. All primer sequences are detailed in **Supplementary Table 1** (Supplementary Material).

### 2.12 Western blotting

Western blotting was performed as described previously (32). Briefly, polyvinylidene difluoride membranes were blocked with 5% skim milk and incubated with primary antibodies targeting the Pik3cb/Akt/mTOR signalling cascade: rabbit anti-p110β (1:1000, K016107P, Solarbio), rabbit anti-phospho-Akt^S473^ (1:1000, K012735RR, Solarbio), rabbit anti-phospho-S6^S240/244^ (phosphorylated ribosomal S6 protein, 1:500, K006232P, Solarbio), rabbit anti-phospho-mTOR^S2448^ (1:1000, K012737RR, Solarbio), rabbit anti-Akt (1:2000, 10176-2-AP, Proteintech, China), rabbit anti-mTOR (1:500, Solarbio) and mouse anti-β-Actin (1:5000, 66009-1-LG, Proteintech, China). Subsequently, the membranes were incubated with goat anti-rabbit or anti-mouse secondary antibodies (1:1000, A0208 or A0216, Beyotime, China) and visualized using enhanced chemiluminescence (Tanon, 5260Muti, Shanghai, China).

### 2.13 Statistical analysis

All experimental data and graphs were analysed using GraphPad Prism 10 software (GraphPad, USA). Quantitative data are presented as mean ± standard deviation (SD). Statistical significance between two groups was determined using Student’s t-test. Comparisons involving three or more groups were analysed using one-way analysis of variance (ANOVA) followed by Tukey’s or Dunnett’s tests for *post-hoc* multiple comparisons. Statistical significance was set at p < 0.05.

## 3 Results

### 3.1 Pik3cb expression is elevated in NOD mouse salivary glands and suppressed by MSC treatment

**Figure 1:**
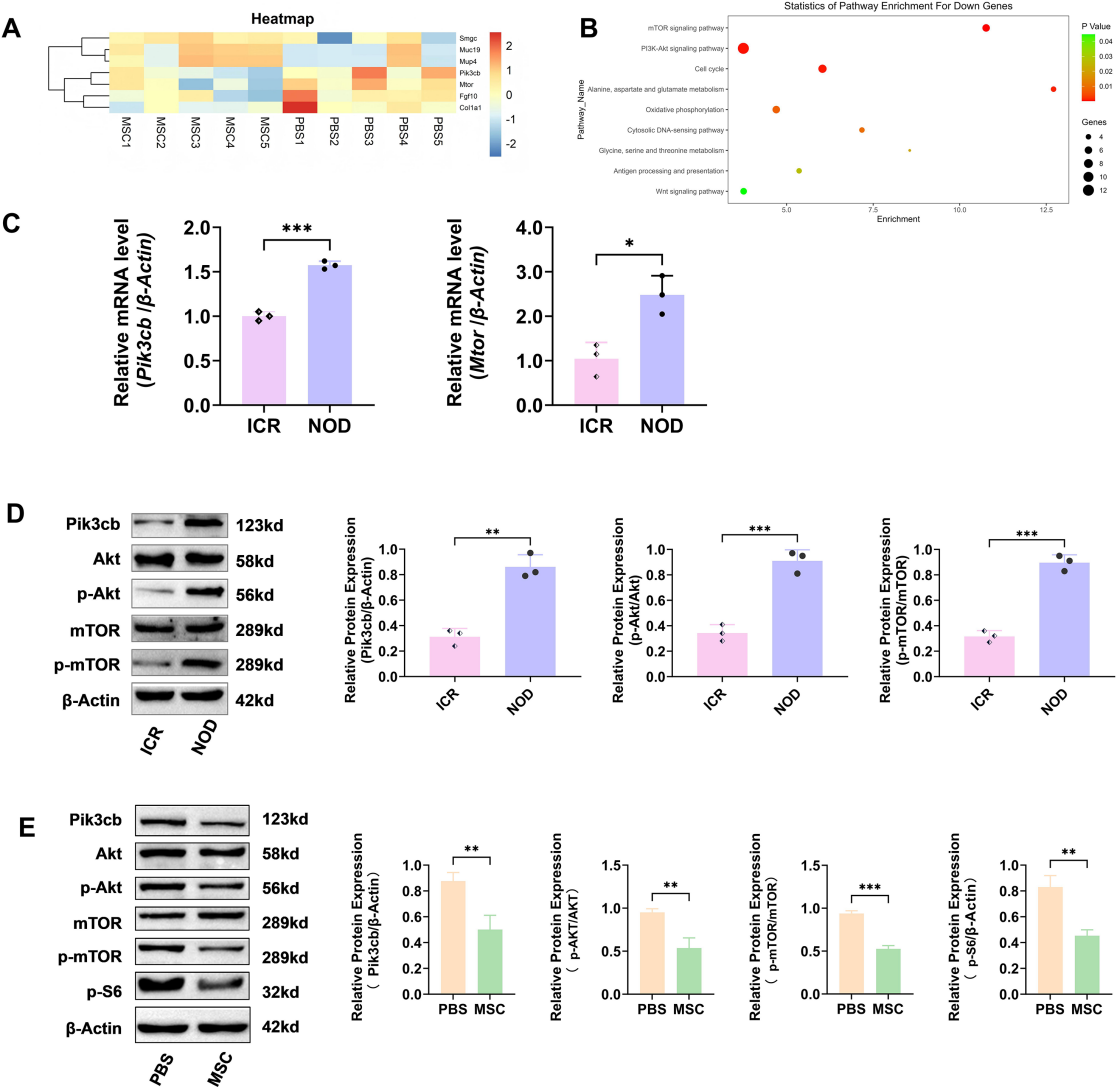
Pik3cb is elevated in NOD mouse salivary glands and suppressed by MSC treatment. **(A)** Heatmap of microarray results showing some important DEGs, including *Smgc*, *Muc19*, *Mup4*, *Pik3cb*, *Mtor*, *Fgf10*, and *Col1a1* in the SMGs of MSC-treated NOD mice *vs* PBS controls (threshold: |log_2_FC| > 1, adjusted p < 0.05, n=5 mice/group). **(B)** KEGG pathway enrichment of DEGs in the MSC group compared to PBS controls (ranked by p-value). **(C)** RT-qPCR analysis of *Pik3cb* and *Mtor* mRNA levels in the SMGs of NOD mice and ICR controls. **(D)** Representative western blots (Left) and quantification (Right) of Pik3cb and its downstream effectors (p-Akt/p-mTOR) in the SMGs of NOD mice *vs* ICR controls. **(E)** Immunoblotting validation of the Pik3cb/Akt/mTOR pathway in MSC group *vs* PBS controls. Left: Representative immunoblot bands. β-Actin served as the loading control. Right: Densitometric quantification of protein levels. Values for Pik3cb and p-S6 were normalized to β-Actin, while p-Akt and p-mTOR were normalized to their respective total proteins. RT-qPCR and Western blotting data are presented as mean ± SD of three independent biological replicates (n=3), *p < 0.05, **p < 0.01, ***p < 0.001. (Student’s t-test). MSC, mesenchymal stem cell; NOD, non-obese diabetic mouse; DEGs, differentially downregulated genes; SMGs, submandibular glands; KEGG, Kyoto Encyclopedia of Genes and Genomes; PBS, phosphate buffered saline; RT-qPCR, real-time quantitative PCR.

We performed genome-wide transcriptomic profiling to compare MSC-treated and PBS-treated NOD mice and elucidate the molecular mechanisms by which MSCs ameliorate SjD-associated immune dysfunction. Our analysis revealed that MSC treatment induced robust transcriptional changes in NOD mouse SMGs, significantly downregulating the key pathogenic mediators *Pik3cb* and *Mtor*, while simultaneously suppressing profibrotic genes (*Fgf10* and *Col1a1*) and upregulating markers of glandular restoration (*Smgc*, *Muc19*, and *Mup4*) (**Figure 1A**). KEGG pathway analysis demonstrated that MSCs coordinately inhibited multiple disease-relevant pathways, notably the mTOR, PI3K-Akt signalling, and cytosolic DNA-sensing pathways (**Figure 1B**). Additionally, network centrality analysis identified *Pik3cb* as a pivotal regulator of MSC therapeutic effects (**Supplementary Figure 1**).

Subsequently, validation experiments demonstrated significantly elevated *Pik3cb* (p < 0.001) and *Mtor* (p < 0.05) mRNA levels in NOD mice compared to those in healthy controls (**Figure 1C**). These findings were further supported at the protein level. Western blotting confirmed the increased protein levels of Pik3cb, p-AKT, and p-mTOR in NOD mice compared to those in controls, all of which were attenuated by MSC therapy (**Figures 1D, E**). Collectively, these data suggest that Pik3cb may serve as an important mediator of the therapeutic effects of MSCs against SjD.

### 3.2 Therapeutic effects of MSCs on glandular function and structure are dependent on Pik3cb suppression

**Figure 2:**
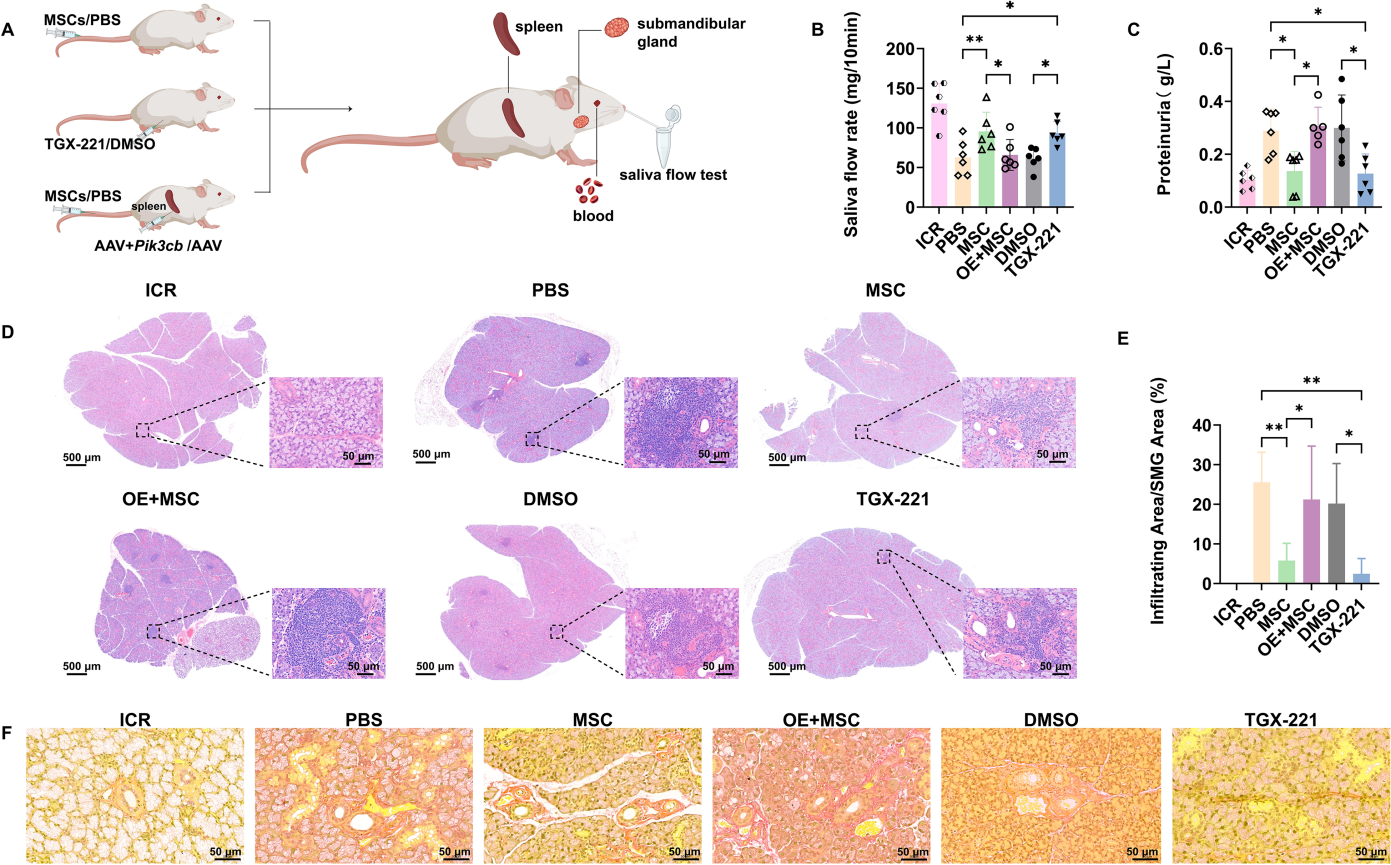
MSC-mediated Pik3cb suppression restores SMG function and architecture in NOD mice. **(A)** Schematic diagram of experimental treatments in NOD mice. **(B)** The saliva flow rate; **(C)** Total proteinuria level. **(D)** H&E-stained SMG sections showing lymphocytic infiltration. **(E)** Quantification of lymphocytic infiltrating area in SMGs (% of total tissue). **(F)** Sirius Red staining showing fibrosis in SMGs; OE + MSC: Pik3cb Overexpression + MSC; Data are presented as mean ± SD (n=6 for B and C, n=3 for E); *p < 0.05, **p < 0.01.

We utilized *in vivo* functional rescue and loss-of-function experiments to functionally validate the hypothesis that Pik3cb suppression mediates the therapeutic effects of MSCs in SjD. First, we confirmed the therapeutic potential of MSCs in NOD mice by administering MSCs or PBS via tail vein injections. The cohorts received either the selective Pik3cb inhibitor TGX-221 or DMSO vehicle i.p. to establish the therapeutic efficacy of Pik3cb inhibition in SjD. Since the spleen is the largest peripheral lymphoid organ and a primary site for B cell maturation and activation. To target the systemic source of pathogenic B cells, in the loss-of-function assay, Pik3cb-overexpressing mice were generated by splenic injection of AAV encoding *Pik3cb*, followed by MSC treatment (OE + MSC group), with parallel controls including OE only and AAV + MSC groups (**Figure 2A**). Successful Pik3cb transgene expression was verified using RT-qPCR and TSA (**Supplementary Figures 2A, D**).

Therapeutic assessment revealed that MSC transplantation and TGX-221 administration significantly restored saliva flow rates in the NOD mice (p < 0.01 for MSC *vs* PBS, and p < 0.05 for TGX-221 *vs* DMSO). Notably, Pik3cb overexpression completely abolished MSC efficacy, resulting in significantly decreased salivary flow rates compared to the MSC or AAV + MSC groups (p < 0.05; **Figure 2B**; **Supplementary Figure 2B**). Additionally, parallel improvements were observed in proteinuria profiles, where both therapies (MSC or TGX-221) normalized proteinuria levels, whereas Pik3cb overexpression prevented therapeutic rescue (p < 0.05; **Figure 2C**).

Subsequently, histopathological assessments demonstrated that MSC and TGX-221 treatment significantly reduced lymphocyte infiltration foci in SMGs compared to that in vehicle controls (p < 0.01 for MSC vs PBS, and p < 0.05 for TGX-221 vs DMSO). Notably, the Pik3cb overexpression groups were refractory to MSC-mediated immunomodulation, exhibiting exacerbated lymphocytic infiltration compared to the MSC and AAV+MSC control groups (**Figures 2D, E**; **Supplementary Figure 2C**). Furthermore, Sirius Red staining confirmed that the antifibrotic effects of both MSC and TGX-221 therapies were similarly compromised by Pik3cb overexpression, as evidenced by obvious collagen deposition in OE + MSC SMGs (**Figure 2F**).

Collectively, these findings establish that MSC-mediated benefits in secretory function and SMG architecture critically depend on the suppression of the Pik3cb pathway. Overexpression of Pik3cb compromises the therapeutic efficacy of MSCs in restoring salivary function and glandular structures.

### 3.3 Pik3cb suppression mediates MSC-induced cytokine recalibration

To delineate the mechanistic role of Pik3cb in MSC-mediated immunomodulation during SjD therapy, we quantified the levels of inflammatory cytokines in the SMGs and spleen tissues using ELISA. Notably, we observed a “pathological focusing” of the autoimmune response in SMGs: despite having fewer total immune cells than the spleen, the SMGs exhibited significantly higher concentrations of IL-4, IL-6 and IFN-γ alongside lower TGF-β1 levels. This distinct compartmentalization reflects the localized “cytokine storm” driven by autoimmune epithelitis in the target gland, contrasting with the more systemic immunoregulatory capacity of the spleen.

**Figure 3:**
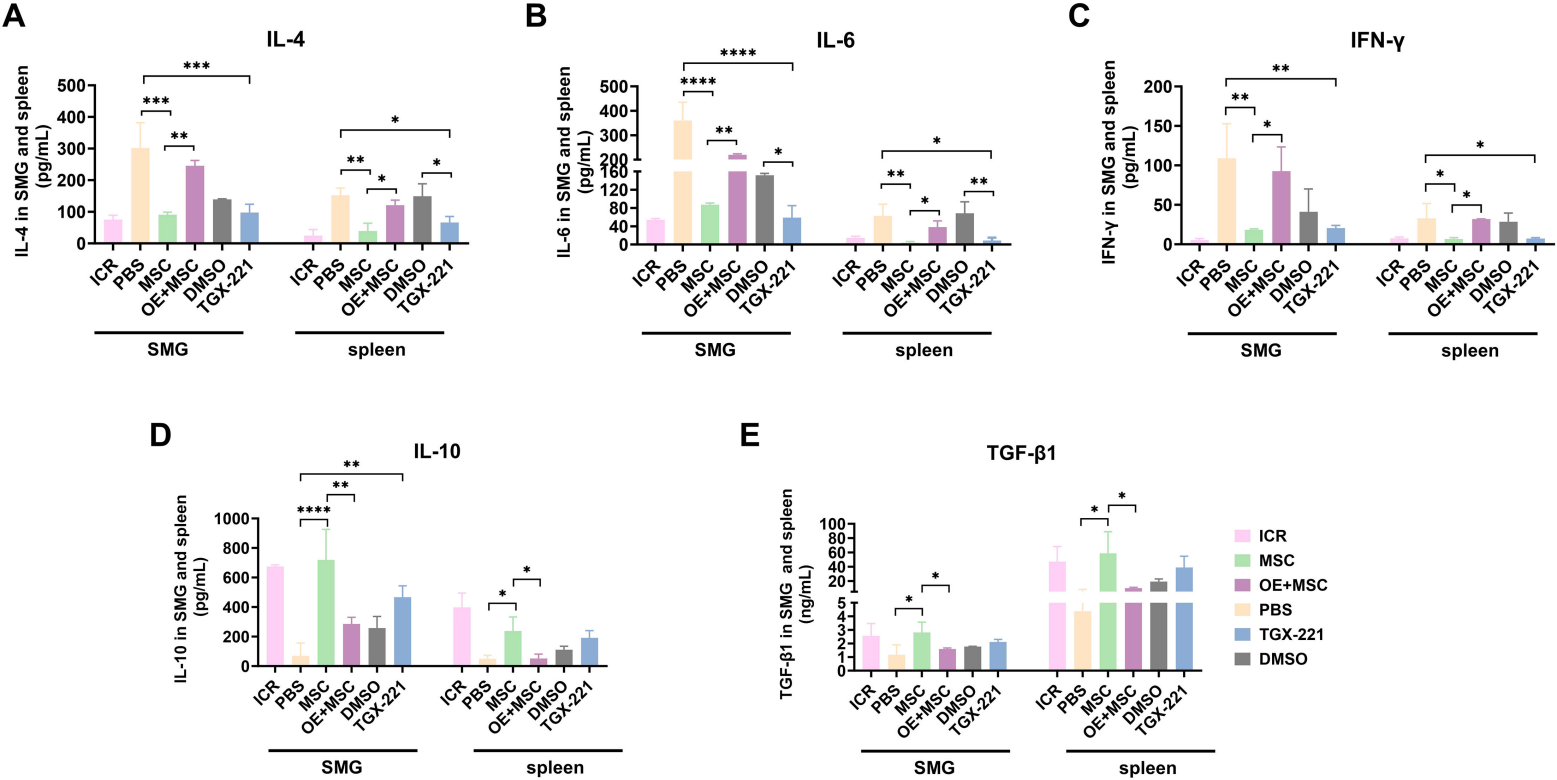
MSC-mediated Pik3cb suppression modulates cytokines in the SMGs and spleens of NOD mice. **(A-C)** Levels of pro-inflammatory cytokines IL-4 **(A)**, IL-6 **(B)**, IFN-γ **(C)** in SMG and spleen tissues assessed by ELISA. **(D, E)** Production of anti-inflammatory cytokines IL-10 **(D)** and TGF-β1 **(E)** in SMGs and spleens. Data are presented as mean ± SD (n=3); *p < 0.05, **p < 0.01, ***p < 0.001, ****p < 0.0001.

Furthermore, we found that both MSC treatment and TGX-221 administration effectively suppressed the secretion of pro-inflammatory cytokines (IL-4, IL-6, and IFN-γ). However, Pik3cb overexpression significantly attenuated MSC-induced suppression of these mediators (**Figures 3A–C**). Conversely, MSCs potently enhanced the levels of anti-inflammatory cytokines (IL-10 and TGF-β1), an effect largely abrogated by Pik3cb overexpression (**Figures 3D, E**). Together, these data demonstrate that MSCs exert their immunomodulatory effects in NOD mice by suppressing Pik3cb signalling and simultaneously curbing pro-inflammatory cytokines while amplifying anti-inflammatory networks.

### 3.4 MSCs inhibit pathogenic B cell and plasma cell activity by targeting the Pik3cb-BAFF axis

**Figure 4:**
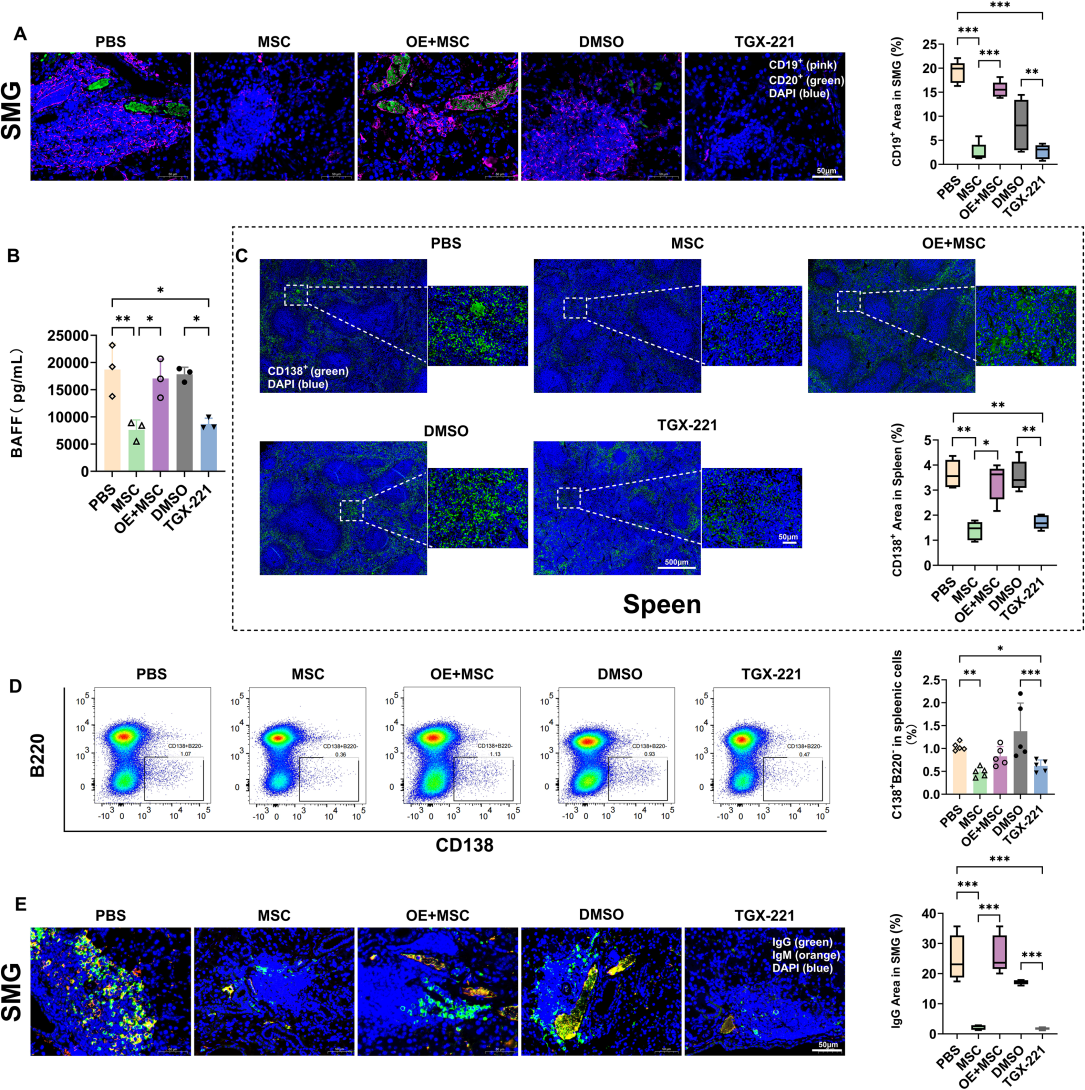
MSCs suppress B cell activation and differentiation in NOD mice by inhibiting Pik3cb. **(A)** Representative TSA images (Left) and quantification (Right) of SMG sections stained for CD19^+^ (pink), CD20^+^ (green) and nucleus (DAPI, blue). MSC-mediated Pik3cb suppression inhibits B cell infiltration in SMGs (Scale bar: 50μm). **(B)** BAFF levels in spleens assessed by ELISA. **(C)** Representative immunofluorescence images (Left) and quantification (Right) of spleen sections show that MSC-mediated Pik3cb suppression decreased plasma cell number (CD138^+^: green, DAPI: blue; Scale bar: 50μm and 500μm). **(D)** Flow cytometry analysis of mature plasma cells (CD138^+^B220^-^) in spleens. Left: representative gating profiles; Right: statistical results of the proportions of CD138^+^ B220^-^ plasma cells in spleens. **(E)** Representative TSA images (Left) and quantification (Right) of SMG sections showing the secretion of immunoglobulins IgG (green) and IgM (orange). Scale bar: 50μm. Data are presented as mean ± SD; *p < 0.05, **p < 0.01, ***p < 0.001. TSA, Tyramide Signal Amplification-based Multiplex Immunofluorescence staining; BAFF, B cell-activating factor.

B cell hyperactivation is a pathological hallmark of SjD and is characterized by excessive antibody production and salivary gland lymphocytic infiltration (4–7). Therefore, we assessed the effects of Pik3cb on B cell subsets and functions. The accumulation of B cells within the SMGs of the PBS and DMSO groups was markedly attenuated by either MSC transplantation or TGX-221 administration. Notably, Pik3cb overexpression effectively reversed this therapeutic effect, leading to a substantial restoration of CD19^+^ B-cell populations (**Figure 4A**). Given that BAFF is essential for B-cell survival and activation, we next investigated whether Pik3cb modulates its secretion in spleens. ELISA results demonstrated that MSC or TGX-221 treatment significantly reduced spleenic BAFF levels (p < 0.01, MSC *vs*. PBS; p < 0.05, TGX-221 *vs*. DMSO); whereas, this suppression was largely bypassed in the Pik3cb-overexpression group, where BAFF remained at levels comparable to disease controls (**Figure 4B**).

We subsequently evaluated the transition of B cells into the plasma cell lineage. IF staining revealed that the expansion of CD138^+^ plasma cells in the spleen was significantly curbed by MSC/TGX-221 treatment, an effect again abrogated by Pik3cb overexpression (**Figure 4C**). Flow cytometric analysis of mature plasma cells (CD138^+^B220^-^) also showed a similar trend. The MSC group exhibited a significantly lower percentage of CD138^+^B220^-^cells than the PBS controls (p < 0.01), which increased with Pik3cb overexpression (**Figure 4D**; **Supplementary Figure 3**).

Finally, to assess the functional output of these cellular changes, we analysed immunoglobulin levels in SMGs. Consistent with the cellular profiles, MSC/TGX-221-mediated suppression of IgG/IgM was similarly negated by Pik3cb overexpression (**Figure 4E**). Collectively, these findings strongly suggest that MSCs suppress pathogenic B-cell activation and plasma cell differentiation by suppressing Pik3cb signalling and subsequently downregulating BAFF.

### 3.5 Pik3cb suppression disrupts Tfh differentiation and CXCL13 production

**Figure 5:**
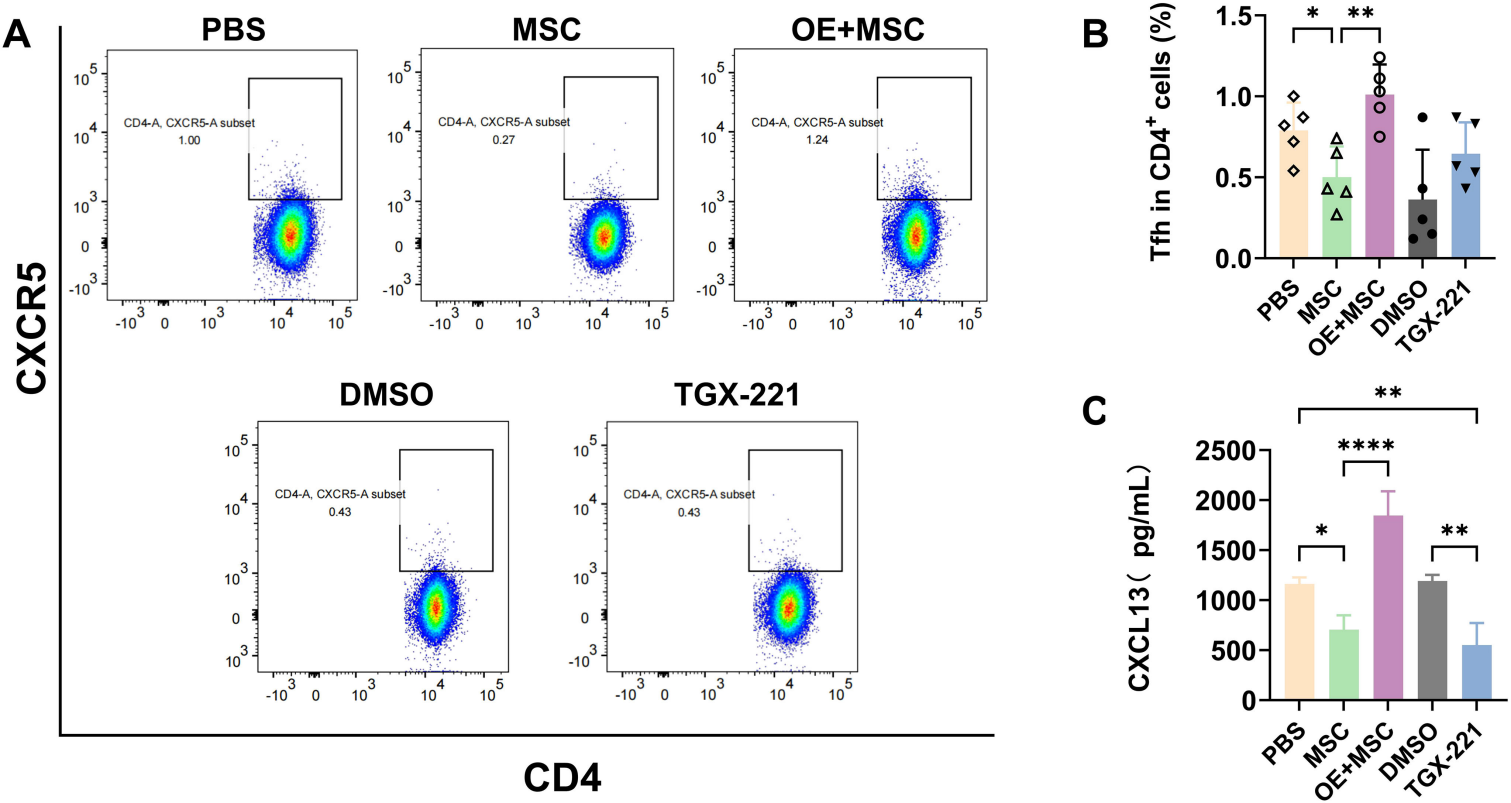
Pik3cb suppresses B cell chemotaxis and activation via Tfh cells and CXCL13. **(A, B)** Flow cytometry analysis of splenic Tfh cells (CD4^+^CXCR5^+^): **(A)** Representative gating profiles of Tfh cells. **(B)** Quantification of Tfh cell frequency in spleens (% of CD4^+^ T cells). **(C)** CXCL13 levels in spleen tissues assessed by ELISA. Data are presented as mean ± SD (n=5 for A and B, n=3 for C); *p < 0.05, **p < 0.01, ****p < 0.0001. Tfh, follicular helper T.

Tfh cells and the CXCL13/CXCR5 chemokine axis are critical regulators of B cell differentiation and ELS formation and play pivotal roles in lymphocyte recruitment (8, 33, 34). Accordingly, flow cytometry data revealed that MSC treatment significantly reduced Tfh cell frequency in the spleen compared to that in PBS controls (p < 0.05). However, this suppression was reversed by Pik3cb overexpression (OE + MSCs group). Notably, the expression level of Tfhs in the OE+MSC group was approximately 2- and 1.5-fold higher than that in the MSC group and TGX-221 groups (**Figures 5A, B**), respectively. A parallel ELISA revealed a similar trend in the modulation of CXCL13 levels in the spleen (**Figure 5C**). These findings with the concomitant reduction in glandular B-cell aggregates and antibody deposition in SMGs (**Figure 4**) collectively suggest that MSCs decrease B lymphocyte recruitment by suppressing Tfh differentiation and CXCL13 production via the inhibition of Pik3cb signalling.

### 3.6 MSCs selectively target B cell Pik3cb/Akt/mTOR signalling in SjD

**Figure 6:**
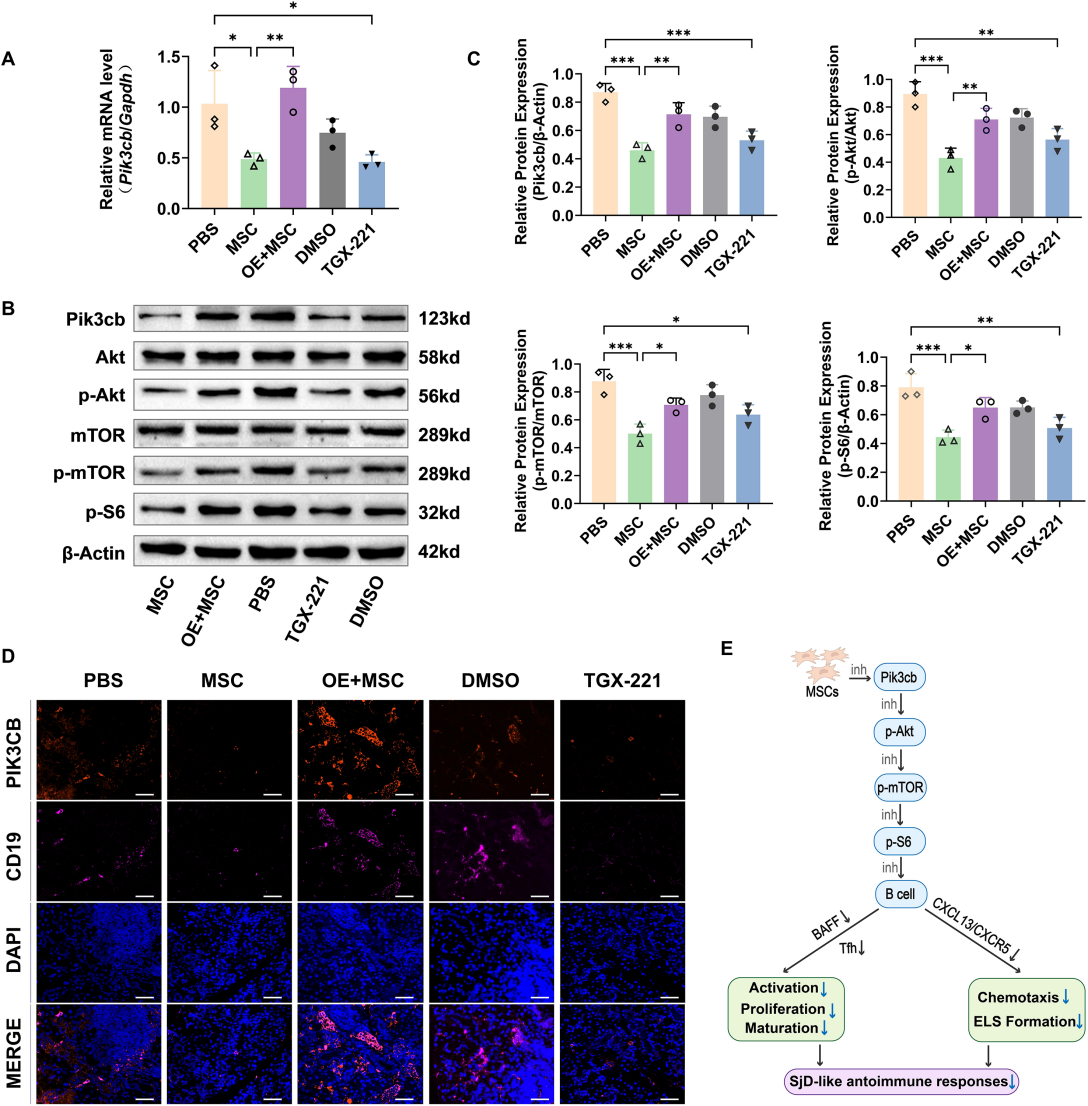
Pik3cb/Akt/mTOR signalling in the SMGs of NOD mice. **(A)** mRNA expression levels of *Pik3cb* in SMG tissues by RT-qPCR. **(B, C)** Western blotting analysis of Pik3cb, Akt, p-Akt (Ser473), mTOR, p-mTOR (Ser2448), and p-S6 (Ser240/244) protein expression in SMGs; **(D)** Representative TSA images of SMG sections showing Pik3cb expression (red) in CD19^+^ B cells (pink). Nuclei counterstained with DAPI (blue). Scale bar: 50 μm; **(E)** Proposed mechanistic schematic: MSCs suppress B cell activation, proliferation, maturation and chemotaxis by inhibiting the Pik3cb/p-Akt/p-mTOR/p-S6 pathway, thereby alleviating SjD-like autoimmune responses (inh: inhibition). Data are presented as mean ± SD (n=3 biological replicates); *p < 0.05, **p < 0.01, ***p < 0.001. SjD, Sjögren disease.

The SMGs from NOD mice exhibited high mRNA levels of *Pik3cb*, indicating disease-associated pathway hyperactivation. Conversely, therapeutic intervention with either MSCs or TGX-221 resulted in a significant downregulation of the corresponding mRNA levels. However, the suppressive effect of MSCs was markedly attenuated by Pik3cb overexpression (p < 0.01; **Figure 6A**). Western blot analysis of Pik3cb, p-Akt, p-mTOR, and p-S6 proteins corroborated these findings, demonstrating that MSC treatment or pharmacological Pik3cb blockade robustly reduced the protein levels of Pik3cb compared to that in PBS controls or OE + MSC mice (**Figures 6B, C**). Representative immunoblot data in spleen pool samples revealed that Pik3cb/Akt/mTOR signalling exhibited an expression trend consistent with the findings in the SMGs (**Supplementary Figure 4**), indicating that MSCs function as potent regulators of the Pik3cb axis across systemic and localized tissues. Notably, TSA identified B lymphocytes (CD19^+^) as the primary cellular compartment exhibiting Pik3cb dysregulation in SjD pathology (**Figure 6D**).

These multimodal findings demonstrate that MSCs exert their therapeutic effects through the selective inhibition of B cell-Pik3cb signalling, subsequently dampening downstream Akt/mTOR/S6 pathway activation in SjD target tissues (**Figure 6E**). The striking consistency across transcriptomic, proteomic, functional, and histopathological analyses suggests that Pik3cb is a mechanistic link mediating MSC efficacy in SjD treatment.

## 4 Discussion

### 4.1 Pik3cb as the novel mechanistic linchpin in MSC-mediated SjD therapy

Our study elucidated the molecular basis underlying the MSC-mediated B-cell immunomodulation in SjD. We demonstrated that Pik3cb suppression mediates a comprehensive array of key therapeutic effects, including the reduction of pathogenic immune cell populations (B cells, plasma cells, and Tfh cells), diminution of inflammatory mediators, attenuation of lymphocytic infiltration, and, critically, functional improvement of salivary secretion. These functional and mechanistic findings collectively establish Pik3cb (encoding PI3Kβ, p110β) as the critical molecular target mediating MSC efficacy in SjD treatment.

The PI3K/Akt/mTOR signalling pathway is a well-established regulator of cell growth, proliferation, differentiation, and survival in various cell types, including immune cells. While other PI3K isoforms, particularly Pik3cd (p110δ) and Pik3cg (p110γ), are canonically linked to immune cell signalling and pathology (35–38), our SjD model highlights the pivotal and unique role of Pik3cb. Functional validation using the Pik3cb-specific inhibitor TGX-221 effectively recapitulated the therapeutic benefits of MSCs, whereas Pik3cb overexpression abolished MSC-mediated therapeutic effects. This establishes the importance of Pik3cb in MSC-based SjD treatment, positioning it distinct from conventional immune modulation paradigms.

These findings are consistent with previous studies that have demonstrated the therapeutic relevance of Pik3cb in inflammatory conditions. Yang et al. (29) compared three PI3K isoform inhibitors in a mouse heart transplantation model. Although p110δ/p110α inhibitors showed stronger immunosuppression, the p110β inhibitor TGX-221 significantly reduced IFN-γ, IL-17A, IL-21 levels and Tfh cell numbers. In this study, we found that IFN-γ and Tfh cells were modulated in a similar manner, consistent with the results of Yang et al. Moreover, the effect of p110β inhibition on Tfh cells is an important mechanism in MSC therapy for SjD. Furthermore, Kulkarni et al. provided compelling evidence that the genetic knockout of Pik3cb markedly ameliorated pathological lesions in autoimmune skin-blistering disease models, further supporting the therapeutic potential of selective Pik3cb inhibition in chronic inflammatory and autoimmune disorders (39, 40).

### 4.2 SjD pathological microenvironment and tumour-like Pik3cb dominance

The critical role of Pik3cb in SjD pathology warrants mechanistic extrapolation beyond the typical autoimmune responses. Studies have revealed that within specific pathological microenvironments such as solid tumours (e.g. prostate cancer and breast cancer), Pik3cb can functionally compensate for Pik3cd or Pik3cg, becoming the dominant isoform that promotes oncogenic signalling and disease progression (41–44). Similarly, chronic inflammation, epithelial cell damage, and tissue remodelling in SjD glands create a unique microenvironment that may render their cellular pathology more akin to certain chronic proliferative disorders (e.g. tumours) rather than a mere antigen-driven autoimmune response (45). Notably, the fact that patients with SjD have a higher lymphoma risk than those with other autoimmune disorders further supports the notion that SjD pathology resembles chronic hyperproliferative diseases, such as malignancies (46–50). Accordingly, approximately 6-10% patients with primary SjD may progress to B-cell non-Hodgkin lymphoma (NHL), likely driven by chronic B-cell activation (51). The accumulated evidence suggests that SjD establishes a tumour-like microenvironment in which Pik3cb emerges as the dominant, survival-sustaining isoform. In contrast, the fact that Pik3cb, instead of other PI3K isoforms, emerged as a core regulatory gene indicates that MSCs may not simply suppress a generalized immune pathway but instead target a disease-induced compensatory mechanism that specifically sustains the survival and hyperproliferation of pathological immune cells.

### 4.3 Pik3cb/Akt/mTOR axis is the metabolic and survival linchpin

Zeng et al. (52) reported significant activation of the PI3K/AKT/mTOR pathway in SjD mice, wherein pharmacological inhibition of this pathway attenuated inflammation and symptoms. In this study, mechanistically, we demonstrated that Pik3cb-mediated Akt/mTOR signalling serves as a metabolic link that sustains pathogenic B cell activity in SjD. Upon Pik3cb activation, p-Akt activates mTOR, a master regulator of B-cell proliferation, clonal expansion, and terminal plasma cell differentiation. This is consistent with established evidence that PI3K-Akt signalling orchestrates multiple aspects of B cell biology, from membrane recruitment upon BCR/CD19 engagement to FOXO-dependent proliferation control (53). Thus, our findings are consistent with those previous report of Zeng et al., further consolidating the role of the PI3K/Akt/mTOR axis as a central therapeutic target in SjD.

The metabolic demands of antibody-secreting plasma cells explain the pathological significance of mTOR in SjD. These cellular “factories” require sustained mTOR activity to fuel massive autoantibody production, evidenced by pS6 (a reliable downstream marker of mTOR) upregulation in SjD patient lymphocytes (51). Our data revealed that MSC administration normalized this pathological metabolic program through Pik3cb inhibition, as evidenced by three relevant findings: (i) Reduced BAFF levels (paralleling observations in SjD patients by Nocturne et al. (54)), a key pathogenic factor in SjD, promoting B cell maturation and proliferation, resulting in autoantibody production (55, 56), (ii) decreased pathogenic B-cell/plasma-cell infiltration, and (iii) diminished IgG/IgM. Collectively, these results suggest that MSC treatment exerts its therapeutic effects by simultaneously targeting the Pik3cb/mTOR metabolic axis and BAFF-mediated survival signals in pathogenic B cells.

### 4.4 Multidimensional efficacy: dismantling lymphoid organization

Furthermore, our findings revealed that MSC-mediated Pik3cb suppression disrupts pathogenic lymphoid organisation by targeting two interdependent pathways. First, Pik3cb suppression significantly reduces CXCL13 production, the key chemokine driving B/Tfh cell migration to the exocrine glands via the CXCL13/CXCR5 axis (57). This chemotactic inhibition prevents the formation of the ELS and subsequent glandular destruction.

MSC treatment also affects Tfh-mediated B-cell activation. Tfh cells are indispensable for the pathogenesis of SjD and serve as master regulators of B cell differentiation and ELS formation. Hyperactive PI3K/AKT/mTOR signalling in SjD preferentially drives CD4+ T cell differentiation toward pathogenic Tfh lineages (58, 59), which sustain B cell maturation through cytokine secretion (IL-21, IL-4, and IL-6) and CD40/CD40L interaction (60–62). As demonstrated in our study, by inhibiting Pik3cb, MSCs effectively interrupt this circuit, reducing Tfh differentiation and CXCR5 expression, thereby disrupting lymphoid organisation.

Collectively, MSCs exert multidimensional therapeutic effects by targeting the Pik3cb/Akt/mTOR cascade to simultaneously dismantle two pathogenic circuits: (i) metabolic and survival support disruption by reducing BAFF and mTOR-dependent survival signals to starve pathogenic B cells/plasma cells and (ii) lymphoid organization impairment by suppressing Tfh and CXCL13 to impair B-cell recruitment and ELS formation. These dual mechanisms ultimately lead to the reversal of glandular pathology in SjD.

### 4.5 Limitations and future perspectives

Despite these significant insights, several limitations of this study warrant consideration. Technically, while the pilocarpine (5 mg/kg) is frequently utilized to elicit saliva in late-stage NOD mice, it occasionally induced transient respiratory distress in rare instances. Future studies could prioritize dose-optimization or the exploration of alternative secretagogues to further refine functional assessments while minimizing physiological stress.

Mechanistically, while we established a link between Pik3cb inhibition and improved SMG pathology, the broader systemic effects—particularly long-term splenic B-cell homeostasis—and the specific MSC-derived factors driving Pik3cb suppression remain to be fully characterized. Finally, although the NOD model closely resembles SjD, validation in diverse primary SjD models and human clinical samples is essential to confirm the translational potential of the Pik3cb/Akt/mTOR axis as a therapeutic target.

## 5 Conclusion

In summary, our study identifies the Pik3cb/Akt/mTOR axis as a potential mechanistic pathway through which MSCs modulate pathogenic B-cell and plasma cell responses in the NOD mouse model. We provides new insights into a previously unrecognized molecular link in SjD-like pathology. Our findings suggest that MSC-mediated suppression of this axis contributes to the amelioration of salivary gland pathology, offering preliminary insights into the immunometabolic regulation of autoimmune epithelitis. While these results underscore Pik3cb as a candidate molecular target, further research remains essential to bridge the gap between this mouse model and human primary SjD. Ultimately, targeting the Pik3cb pathway, perhaps in conjunction with established B-cell-targeted therapies, may provide a basis for developing more refined therapeutic strategies for SjD.

## Data Availability

The original contributions presented in the study are included in the article/supplementary material. Further inquiries can be directed to the corresponding author.
