# Supplementary material for: Mesenchymal stem cells ameliorate Sjögren disease by suppressing B cells through the Pik3cb/Akt/mTOR pathway
Source: Front Immunol. 2026 Mar 12;17:1761950. doi: 10.3389/fimmu.2026.1761950 (PMC13017803; doi:10.3389/fimmu.2026.1761950)
Supplement: Supplementary file 1 [file Table1.docx]

Supplementary Material


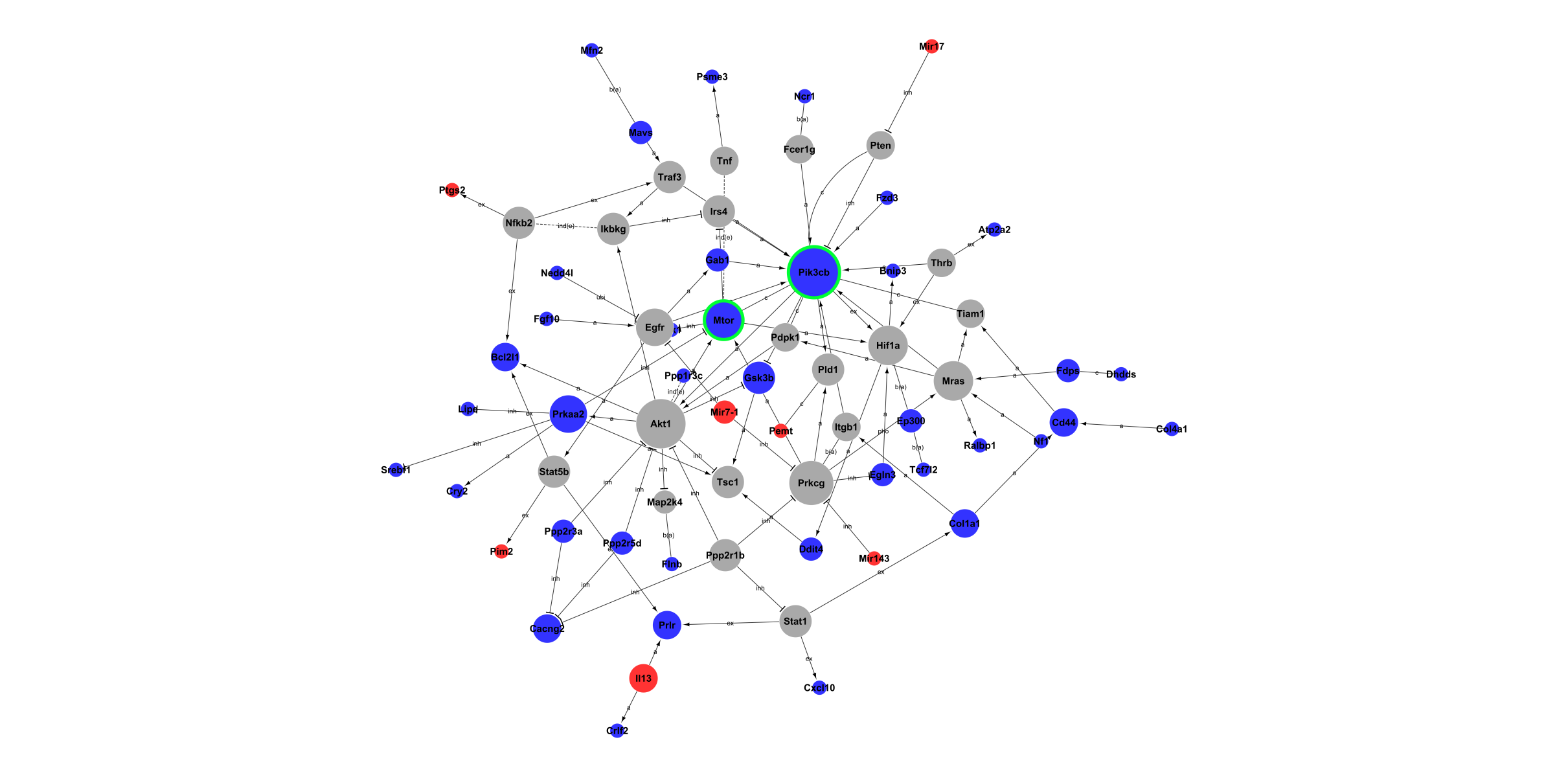


**Supplementary Figure 1.** Key signaling network identifies *Pik3cb* as the key node mediating MSC therapeutic effects in SjD. Node centrality and edge weight reflect predictive interaction strength in the context of MSC treatment.


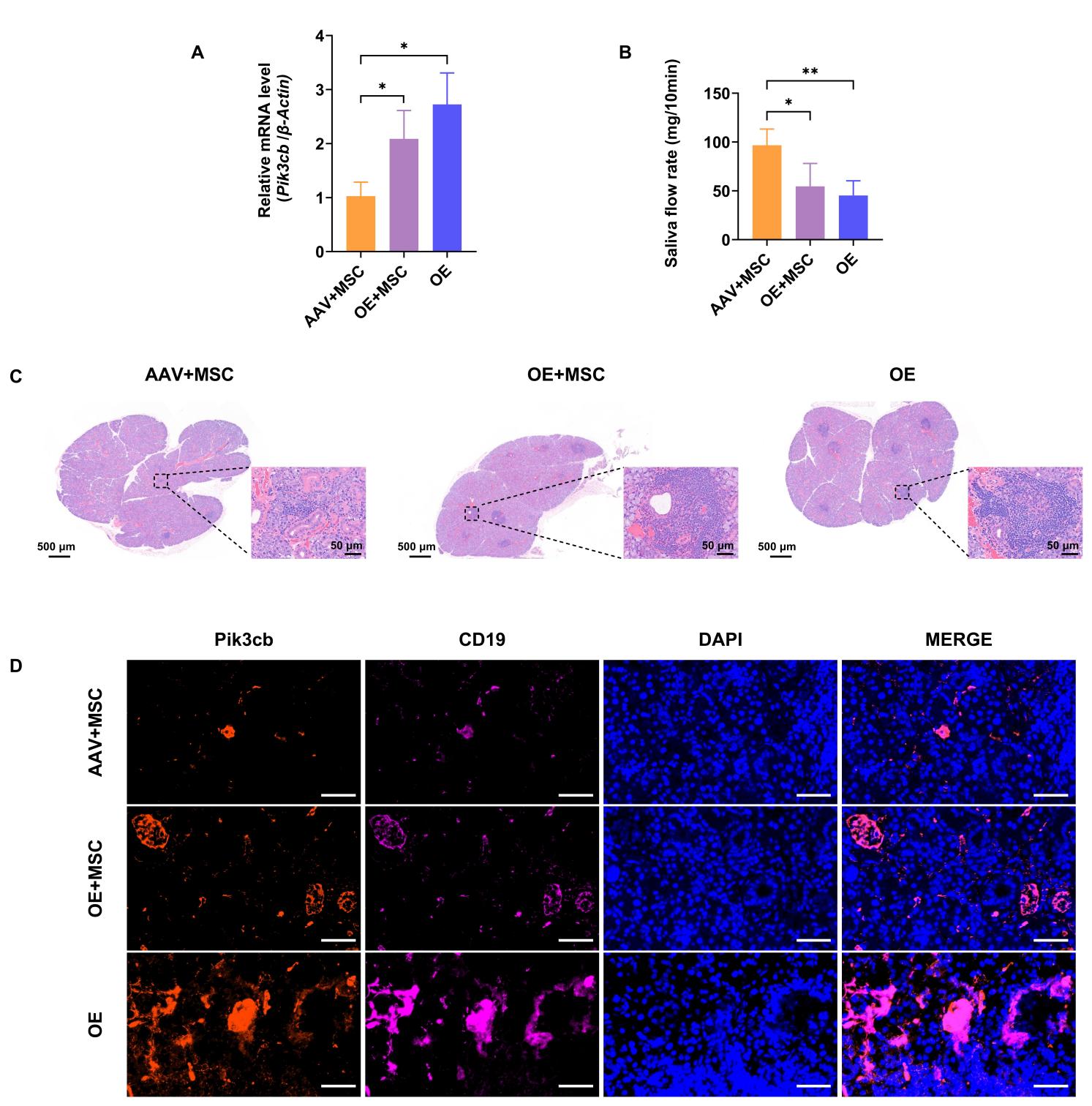


**Supplementary Figure 2.** Pik3cb overexpression exacerbates salivary gland dysfunction and disruption in NOD mice. (A) mRNA expression levels of *Pik3cb* in SMG tissues by RT-qPCR confirming successful overexpression; (B) The saliva flow rate is reduced in Pik3cb-overexpressing mice; (C) H&E-stained SMG sections showing aggravated lymphocytic infiltration in Pik3cb-overexpressing mice; (D) Representative TSA staining of SMG sections showing Pik3cb expression (red) in CD19^+^ B cells (pink). Nuclei stained with DAPI (blue). Scale bar: 50 μm. Data are presented as mean ± SD (n=5); *p < 0.05, **p < 0.01.


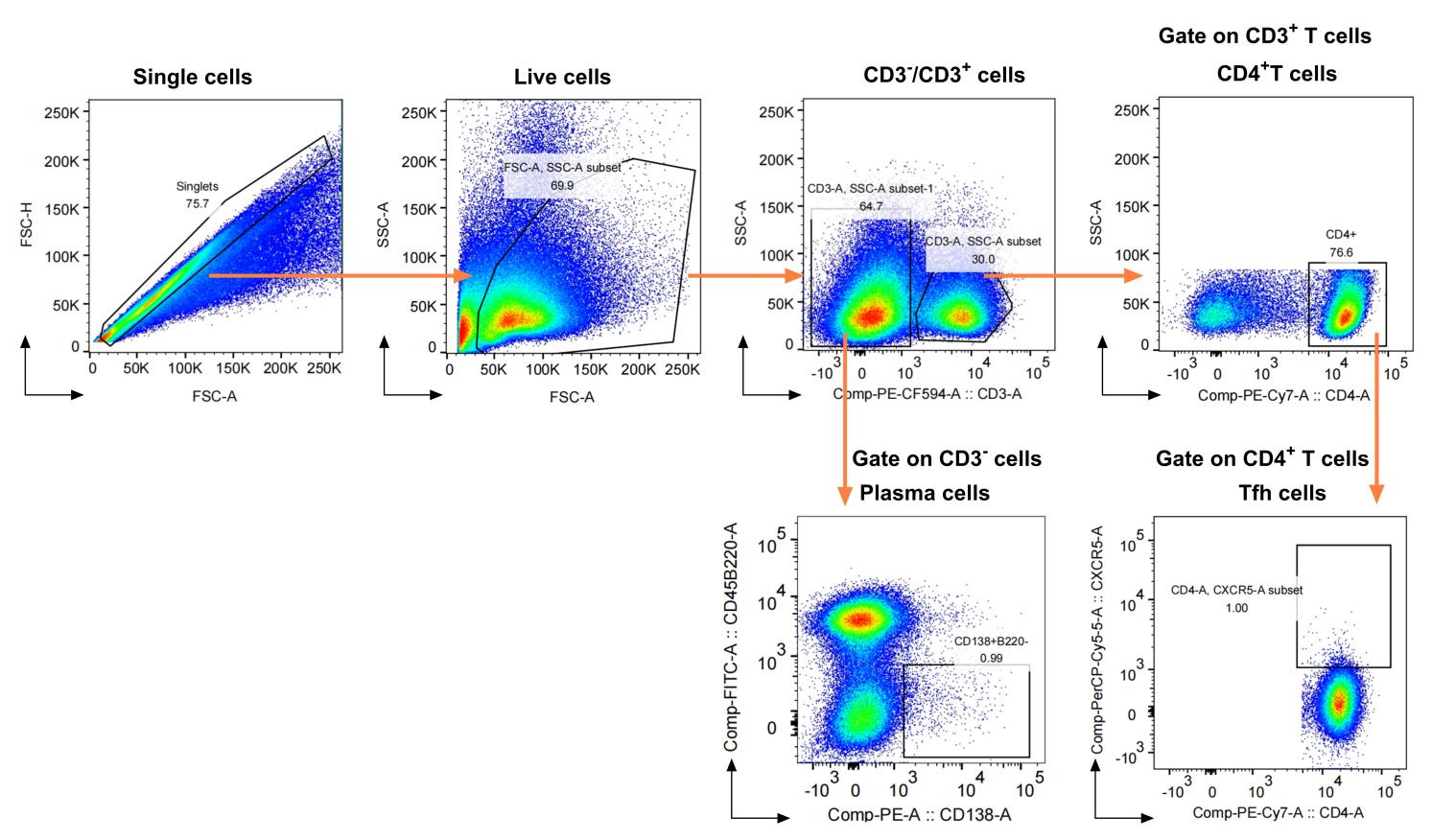


**Supplementary Figure 3.** Flow cytometry gating strategy for identification of lymphocyte subsets in NOD mouse spleen tissues. The populations of interest were sequentially identified using the following established gating strategy, starting from the total cell suspension. Single Cells: Excluded debris and aggregates using FSC and SSC parameters. Live cells: Gated on the characteristic FSC/SSC profile within the single-cell population. CD3^-^/CD3^+^ Cells: Gated on the live cells. CD4^+^ T Cells: Gated on CD3^+^ T lymphocytes. Tfh Cells (Follicular Helper T Cells): Gated as CXCR5^+^ CD4^+^ T cells within the CD4^+^ T population. Mature Plasma Cells: Identified as the CD138^+^ population that is negative for the B cell marker B220, and gated on CD3^-^ cells.


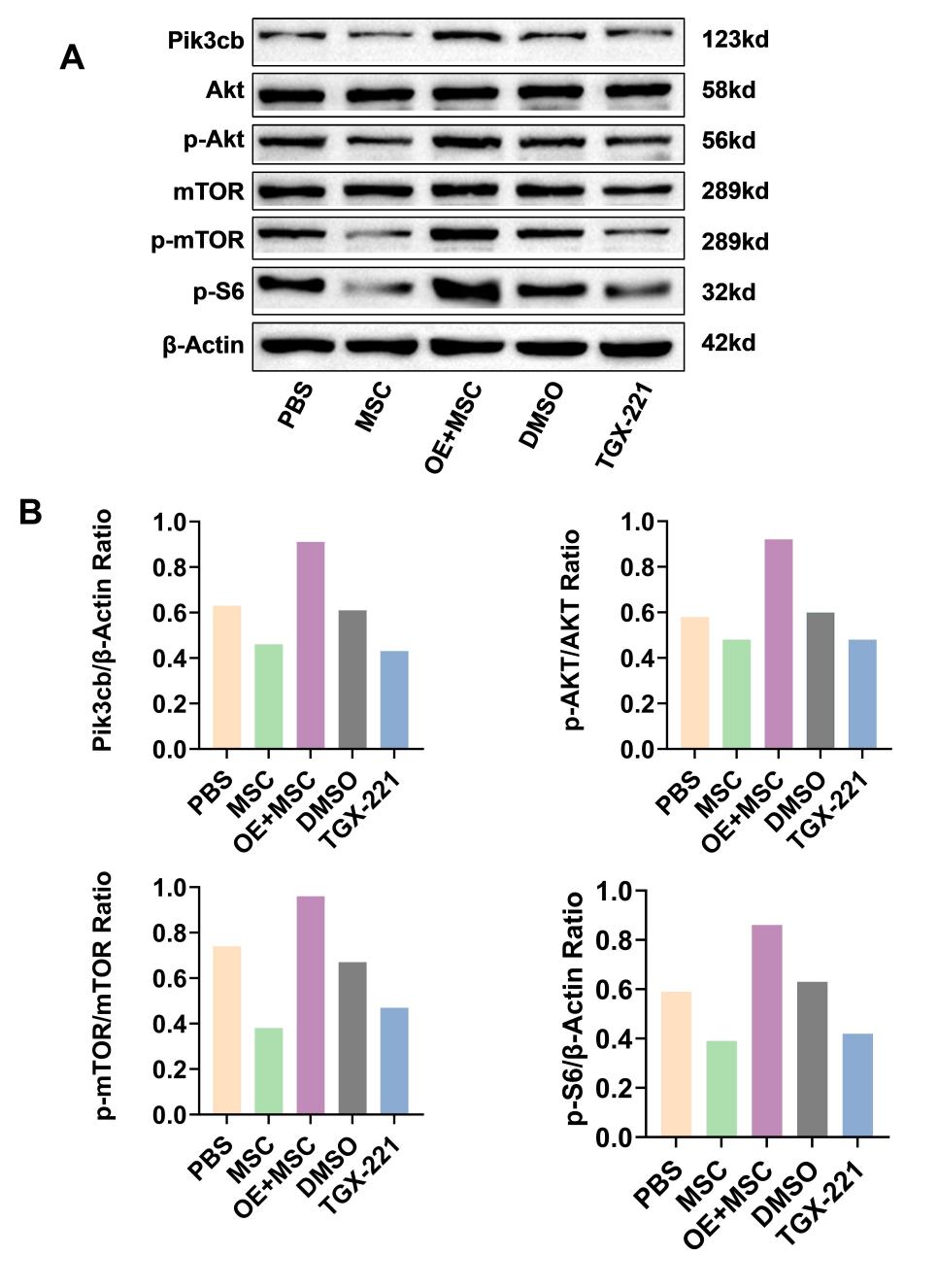


**Supplementary Figure 4.** Immunoblot analysis of the Pik3cb/Akt/mTOR pathway in pooled splenic samples. (A) Immunoblot bands display the protein expression levels of Pik3cb, total Akt, p-Akt (Ser473), total mTOR, p-mTOR (Ser2448), and p-S6 (Ser240/244) in the spleen tissues. Each lane contains pooled protein extracts from 3 mice per group to ensure a consensus biological profile. β-Actin was used as an internal loading control. (B) Relative quantification via densitometry. Bars represent the ratio of target proteins normalized to β-Actin (or phospho-protein/total protein ratio). Note: Data represent a single analysis of pooled samples to validate the systemic trend. Due to the pooling of samples (n=3 per group), no statistical analysis was performed for this supplementary validation.

**Supplementary Table 1**. Primer sequences of genes for RT-qPCR.

| Gene | Primer sequence (5'to3') |
| --- | --- |
| *Pik3cb-F* | TGGCTTGGACCTGCGGATG |
| *Pik3cb-R* | GAATGTCAGCGATTGTCTCAGAGG |
| *Mtor-F* | CCGCTACTGTGTCTTGGCAT |
| *Mtor-R* | CAGCTCGCGGATCTCAAAGA |
| *Akt1-F* | TCAGGATGTGGATCAGCGAGAGTC |
| *Akt1-R* | AGGCAGCGGATGATAAAGGTGTTG |
| *β-Actin-F* | GTGACGTTGACATCCGTAAAGA |
| *β-Actin-R* | GCCGGACTCATCGTACTCC |
| *Gapdh-F* | TGGCCTTCCGTGTTCCTAC |
| *Gadph-R* | GAGTTGCTGTTGAAGTCGCA |
